# Detection and measure of genuine tripartite entanglement with partial transposition and realignment of density matrices

**DOI:** 10.1038/s41598-017-17585-7

**Published:** 2017-12-08

**Authors:** Ming Li, Jing Wang, Shuqian Shen, Zhihua Chen, Shao-Ming Fei

**Affiliations:** 1 College of the Science, China University of Petroleum, Qingdao, 266580 P. R. China; 2 Department of Science, Zhijiang college, Zhejiang University of Technology, Hangzhou, 310024 P. R. China; 3 School of Mathematical Sciences, Capital Normal University, Beijing, 100048 P. R. China; 4 Max-Planck-Institute for Mathematics in the Sciences, Leipzig, 04103 Germany

## Abstract

It is challenging task to detect and measure genuine multipartite entanglement. We investigate the problem by considering the average based positive partial transposition(PPT) criterion and the realignment criterion. Sufficient conditions for detecting genuine tripartite entanglement are presented. We also derive lower bounds for the genuine tripartite entanglement concurrence with respect to the conditions. While the PPT criterion and the realignment criterion are powerful for detecting bipartite entanglement and for providing lower bounds of bipartite concurrences, our results give an effective operational way to detect and measure the genuine tripartite entanglement.

## Introduction

Quantum entanglement is recognized as a remarkable resource in the rapidly expanding field of quantum information science, with various applications^1^. A multipartite quantum state that is not separable with respect to any bi-partition is said to be genuinely multipartite entangled(GME)^2^, which is one of the important type of entanglement, and offers significant advantage in quantum tasks comparing with bipartite entanglement^3^. In particular, it is the basic ingredient in measurement-based quantum computation^4^, and is beneficial in various quantum communication protocols, including secret sharing^5,6^, extreme spin squeezing^7^, high sensitivity in some general metrology tasks^8^, quantum computing using cluster states^9^, and multiparty quantum network^10^. Although its significance, detecting and measuring such kind of entanglement turns out to be quite difficult. To certify GME, an abundance of linear and nonlinear entanglement witnesses^11–19^, generalized concurrence for multi genuine entanglement^20–23^, and Bell-like inequalities^24^entanglement witnesses were derived (see e.g. reviews^2,25^) and a characterisation in terms of semi-definite programs (SDP) was developed^26,27^. Nevertheless, the problem remains far from being satisfactorily solved.

For bipartite systems, Peres in^28^ has presented a much stronger separability criterion, which is called positive partial transpose (PPT) criterion. It says that if *ρ*_*AB*_ is separable, then the partial transposition ${\rho }_{AB}^{{T}_{B}}$ with matrix elements defined as: ${({\rho }_{AB}^{{T}_{B}})}_{ij,kl}={\rho }_{il,kj}$ is a density operator (i.e. has nonnegative spectrum). It has interpretation as a partial time reversal^29^. There is yet another strong class of criteria based on linear contractions on product states. They stem from the new criterion discovered in^30,31^ called computable cross norm criterion or matrix realignment criterion(CCNR) which is operational and independent on PPT test^28^. In terms of matrix elements it can be stated as follows: if the state *ρ*_*AB*_ is separable then the matrix ${\mathcal R} (\rho )$ with elements ${\mathcal R} {(\rho )}_{ij,kl}$ = *ρ*_*ik*_,_*jl*_ has trace norm not greater than one, i.e. ||${\mathcal R} (\rho )$||_*KF*_ ≤ 1. Quite remarkably, the realignment criterion has been found to be able to detect some PPT entangled states^30,31^ and to be useful for construction of some nondecomposable maps. It also provides nice lower bound on concurrence^32^. Further more, a necessary and sufficient criterion of the local unitary equivalence for general multipartite states based on matrix realignment has been presented in^33^.

In this manuscript, we investigate the detection of GME for arbitrary tripartite quantum systems. We will derive an effective criterion based on PPT and CCNR. A lower bound for GME concurrence will be also obtained. We then compute examples to show the effectiveness of our results.

## Results

In the following, we present a criterion to detect GME for tripartite qudits systems by using the PPT and CCNR criteria. A lower bound for GME concurrence of tripartite quantum systems will be also obtained. We start with some definitions and notations.

Let ${H}_{i}^{d}$, *i* = 1, 2, 3, denote *d*-dimensional Hilbert spaces. A tripartite state $\rho \in {H}_{1}^{d}\otimes {H}_{2}^{d}\otimes {H}_{3}^{d}$ can be expressed as $\rho =\sum {p}_{\alpha }|{\psi }_{\alpha }\rangle \langle {\psi }_{\alpha }|$, where 0 < *p*_*α*_ ≤ 1, ∑*p*_*α*_ = 1, $|{\psi }_{\alpha }\rangle \in {H}_{1}^{d}\otimes {H}_{2}^{d}\otimes {H}_{3}^{d}$ are normalized pure states. If all $|{\psi }_{\alpha }\rangle$ are biseparable, namely, either $|{\psi }_{\alpha }\rangle =|{\phi }_{\alpha }^{1}\rangle \otimes |{\phi }_{\alpha }^{23}\rangle$ or $|{\psi }_{\beta }\rangle =|{\phi }_{\beta }^{2}\rangle \otimes |{\phi }_{\beta }^{13}\rangle$ or $|{\psi }_{\gamma }\rangle =|{\phi }_{\gamma }^{3}\rangle \otimes |{\phi }_{\gamma }^{12}\rangle$, where $|{\phi }_{\gamma }^{i}\rangle$ and $|{\phi }_{\gamma }^{ij}\rangle$ denote pure states in ${H}_{i}^{d}$ and ${H}_{i}^{d}\otimes {H}_{j}^{d}$ respectively, then *ρ* is said to be bipartite separable. Otherwise, *ρ* is called genuine multipartite entangled.

Define that $M(\rho )=\frac{1}{3}(\Vert {\rho }^{{T}_{1}}\Vert +\Vert {\rho }^{{T}_{2}}\Vert +\Vert {\rho }^{{T}_{3}}\Vert ),$ $N(\rho )=\frac{1}{3}(\Vert {R}_{1|23}(\rho )\Vert +\Vert {R}_{2|13}(\rho )\Vert +\Vert {R}_{3|12}(\rho )\Vert ),$ where *T*_*i*_ s are the partial transposition over the *i* th subsystem, *i* = 1, 2, 3 and *R*_*i*|*jk*_ stands for the bipartite realignment with respect to subsystem *i* and subsystems *jk*, *i*, *j*, *k* = 1, 2, 3. $\Vert \cdot \Vert$ denotes the trace norm of a matrix.

To derive GME criterion, we first obtain the following lemma.

***Lemma:*** Let *d* = min{*m*, *n*}. For a bipartite quantum state $|\phi \rangle \in {H}_{A}^{m}\otimes {H}_{B}^{n},$ we have $\Vert {(|\phi \rangle \langle \phi |)}^{{T}_{A}}\Vert \le d$, and $\Vert {R}_{A|B}(|\phi \rangle \langle \phi |)\Vert \le d$.

**Proof**. By Schmidt decomposition, we set $|\phi \rangle =\sum _{i\mathrm{=1}}^{d}\sqrt{{u}_{i}}|ii\rangle$ with $\sum _{i=1}^{d}{u}_{i}=1,{u}_{i}\ge 0.$ By the Cauchy-Schwarz inequality one computes

1 $$\Vert {(|\phi \rangle \langle \phi |)}^{{T}_{A}}\Vert =\Vert {R}_{A|B}(|\phi \rangle \langle \phi |)\Vert ={(\sum _{i}\sqrt{{u}_{i}})}^{2}\le d{(\sum _{i}{u}_{i})}^{2}=d\mathrm{.}$$

Then we are ready to show the theorems.      ■

**Theorem 1:** Let $\rho \in {H}_{123}={H}_{1}^{d}\otimes {H}_{2}^{d}\otimes {H}_{3}^{d}$ be a tripartite qudits quantum state. If *ρ* is bipartite separable, then $\max \,\{M(\rho ),N(\rho )\}\le \frac{1+2d}{3}$ must hold. Or equivalently, if $\max \,\{M(\rho ),N(\rho )\} > \frac{1+2d}{3},$ then *ρ* is GME.

See Methods for the proof of theorem 1.

The GME concurrence for tripartite quantum systems, which is defined as follows, is proved to be a well defined measure^20,21^. For a pure state $|\psi \rangle \in {H}_{1}^{d}\otimes {H}_{2}^{d}\otimes {H}_{3}^{d}$, the GME concurrence is defined by

$${C}_{GME}(|\psi \rangle )=\sqrt{\min \,\{1-tr({\rho }_{1}^{2}),1-tr({\rho }_{2}^{2}),1-tr({\rho }_{3}^{2})\}},$$

where *ρ*_*i*_ is the reduced matrix for the *i* th subsystem. For mixed state $\rho \in {H}_{1}^{d}\otimes {H}_{2}^{d}\otimes {H}_{3}^{d}$, the GME concurrence is then defined by the convex roof

2 $${C}_{GME}(\rho )=\,\min \sum _{\{{p}_{\alpha },|{\psi }_{\alpha }\rangle \}}{p}_{\alpha }{C}_{GME}(|{\psi }_{\alpha }\rangle ).$$

The minimum is taken over all pure ensemble decompositions of *ρ*. Since one has to find the optimal ensemble to do the minimization, the GME concurrence is hard to compute. In the following we derive an effective lower bound for GME concurrence in terms of the PPT criterion and the CCNR criterion.

**Theorem 2:** Let $\rho \in {H}_{123}={H}_{1}^{d}\otimes {H}_{2}^{d}\otimes {H}_{3}^{d}$ be a tripartite qudits quantum state. Then one has

3 $${C}_{GME}(\rho )\ge \frac{1}{\sqrt{d(d-1)}}(\max \,\{M(\rho ),N(\rho )\}-\frac{1+2d}{3}).$$

See Methods for the proof of theorem 2.

### Applications

The following two examples show that the criterion and the lower bound of GME concurrence above are much effective for detecting and measuring GME in tripartite quantum systems.

**Example 1:** Consider quantum state $\rho \in {H}_{1}^{3}\otimes {H}_{2}^{3}\otimes {H}_{3}^{3},$ $\rho =\frac{1-x}{27}I+x|\phi \rangle \langle \phi |,$ where $|\phi \rangle =\frac{1}{\sqrt{3}}(|000\rangle +$ $|111\rangle +|222\rangle )$ is the GHZ state. By Theorem 1 in^13^ we can detect GME for 0.894427 < *x* ≤ 1. Using the Theorem 1 in this manuscript, we compute $\max \,\{M(\rho ),N(\rho )\}=\frac{1}{9}(8+10x+|10x-1|)$. Thus GME is detected for 0.7< *x* ≤1.

**Figure 1:**
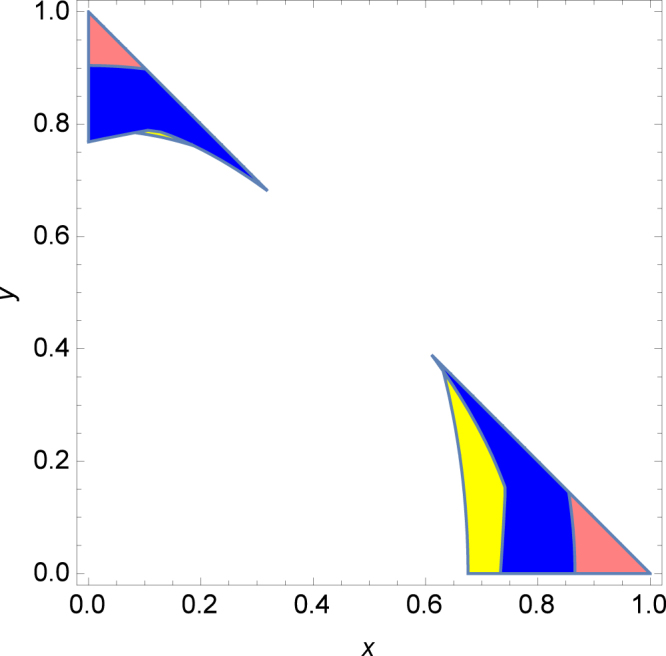
GME Detected by Vicente criterion (pink region by Theorem 1 and yellow region by Theorem 2 in^13^) and by the theorem 1 in this manuscript(blue region).

**Example 2:** We consider the mixture of the GHZ state and W state in three-qubit quantum systems $\rho =\frac{1-x-y}{8}I+x|GHZ\rangle \langle GHZ|+y|W\rangle \langle W|$, where $|GHZ\rangle =\frac{1}{\sqrt{2}}(|000\rangle +|111\rangle )$ and $|W\rangle =\frac{1}{\sqrt{3}}(|001\rangle +$ $|010\rangle +|100\rangle )$. As shown in Fig. 1, our criterion detect some GME(blue region) that can not be detected by Vicente criteria.

**Figure 2:**
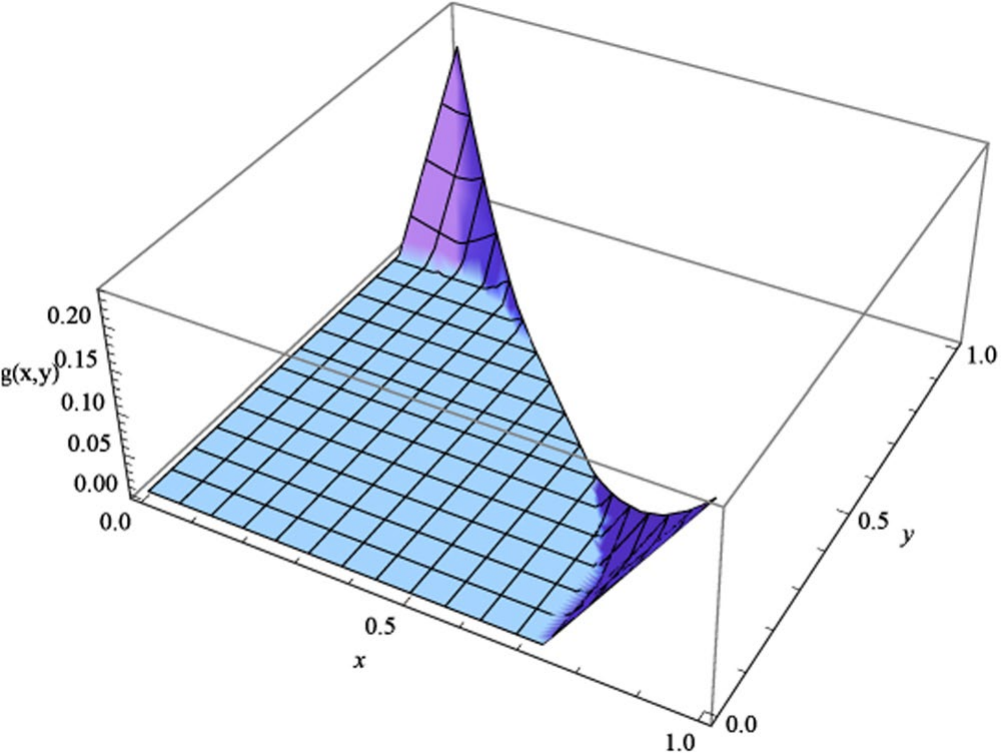
The lower bound of GME concurrence for *ρ* in example 2. *g*(*x*, *y*) stands for the lower bound.

The lower bound of GME concurrence in Theorem 2 for *ρ* is computed to be

$$\begin{array}{rcl}g(x,y) & = & \mathrm{(1/(24}\sqrt{2}))(-40+3\sqrt{{(-1-3\alpha +\beta )}^{2}}+6\sqrt{{(-1+\alpha +\beta )}^{2}}+\sqrt{{\mathrm{(3}-3\alpha +13\beta )}^{2}}\\  &  & +\sqrt{9+153{\alpha }^{2}+6\beta +17{\beta }^{2}-6\alpha \mathrm{(3}+\beta )-8\sqrt{{\mathrm{(3}-3\alpha +\beta )}^{2}\mathrm{(9}{\alpha }^{2}+{\beta }^{2})}}\\  &  & +\sqrt{9+153{\alpha }^{2}+6\beta +17{\beta }^{2}-6\alpha \mathrm{(3}+\beta )+8\sqrt{{\mathrm{(3}-3\alpha +\beta )}^{2}\mathrm{(9}{\alpha }^{2}+{\beta }^{2})}}\\  &  & +\sqrt{9+45{\alpha }^{2}-18\alpha (-1+\beta )-18\beta +137{\beta }^{2}-12\sqrt{{\mathrm{(1}+\alpha -\beta )}^{2}\mathrm{(9}{\alpha }^{2}+32{\beta }^{2})}}\\  &  & +\sqrt{9+45{\alpha }^{2}-18\alpha (-1+\beta )-18\beta +137{\beta }^{2}+12\sqrt{{\mathrm{(1}+\alpha -\beta )}^{2}\mathrm{(9}{\alpha }^{2}+32{\beta }^{2})}})\end{array}$$

as ploted in Fig. 2.

## Discussions

It is a basic and fundamental question in quantum information theory to detect and measure GME. In this manuscript we have presented a GME criterion based on the PPT and Realignment criteria. A lower bound of GME concurrence for tripartite quantum system has also been obtained. Examples show that our criterion is independent of Vicente criteria and can detect more genuine entangled quantum states. Our results are derived by average based PPT and CCNR criteria. One can construct more effective criteria to detect GME and lower bounds of GME concurrence by taking the average of the correlation matrices or covariance matrices and so on. It is also of interesting to investigate the implementation of the criterion with measurements or to extend the results to systems consisting of more than three parties.

## Methods

**Proof of Theorem 1** Lets consider a pure state $\rho =|\psi \rangle \langle \psi |$ first. Assume that $|\psi \rangle \in {H}_{123}={H}_{1}^{d}\otimes {H}_{2}^{d}\otimes {H}_{3}^{d}$ be bipartite separable, which will be in one of the following three forms: |*ψ*〉 = |*φ*_1_〉 ⊗ |*φ*_23_〉, |*ψ*〉 = |*φ*_2_〉 ⊗ |*φ*_13_〉, or $|\psi \rangle =|{\phi }_{3}\rangle \otimes |{\phi }_{12}\rangle$. If $|\psi \rangle =|{\phi }_{1}\rangle \otimes |{\phi }_{23}\rangle ,$ then by using the first two equations in (1) we have

$$\begin{array}{rcl}\Vert {\rho }^{{T}_{1}}\Vert  & = & \Vert {R}_{\mathrm{1|23}}(\rho )\Vert =\Vert {(|{\phi }_{1}\rangle \langle {\phi }_{1}|)}^{{T}_{1}}\otimes |{\phi }_{23}\rangle \langle {\phi }_{23}|\Vert =\mathrm{1;}\\ \Vert {\rho }^{{T}_{2}}\Vert  & = & \Vert {R}_{\mathrm{2|13}}(\rho )\Vert =\Vert |{\phi }_{1}\rangle \langle {\phi }_{1}|\Vert \cdot \Vert {(|{\phi }_{23}\rangle \langle {\phi }_{23}|)}^{{T}_{2}}\Vert =\Vert {(|{\phi }_{23}\rangle \langle {\phi }_{23}|)}^{{T}_{2}}\Vert \le d;\\ \Vert {\rho }^{{T}_{3}}\Vert  & = & \Vert {R}_{\mathrm{3|12}}(\rho )\Vert =\Vert |{\phi }_{1}\rangle \langle {\phi }_{1}|\Vert \cdot \Vert {(|{\phi }_{23}\rangle \langle {\phi }_{23}|)}^{{T}_{2}}\Vert \le d\mathrm{.}\end{array}$$

Similarly, one has

$$\Vert {\rho }^{{T}_{1}}\Vert =\Vert {R}_{1|23}(\rho )\Vert \le d;\Vert {\rho }^{{T}_{2}}\Vert =\Vert {R}_{2|13}(\rho )\Vert =1;\Vert {\rho }^{{T}_{3}}\Vert =\Vert {R}_{3|12}(\rho )\Vert \le d$$

for $$|\psi \rangle =|{\phi }_{2}\rangle \langle {\phi }_{13}|$$ and

$$\Vert {\rho }^{{T}_{1}}\Vert =\Vert {R}_{1|23}(\rho )\Vert \le d;\Vert {\rho }^{{T}_{2}}\Vert =\Vert {R}_{2|13}(\rho )\Vert \le d;\Vert {\rho }^{{T}_{3}}\Vert =\Vert {R}_{3|12}(\rho )\Vert =1$$

for $|\psi \rangle =|{\phi }_{3}\rangle \langle {\phi }_{12}|$ respectively. Thus for any type bipartite separable pure quantum state, we always have $M(\rho )\le \frac{1+2d}{3},$ and $N(\rho )\le \frac{1+2d}{3}.$

For mixed bipartite separable state *ρ*, by using the convex property of *M*(*ρ*) and *N*(*ρ*) we obtain

4 $$M(\rho )\le \sum {p}_{\alpha }M(|{\psi }_{\alpha }\rangle \langle {\psi }_{\alpha }|)\le \frac{1+2d}{3},$$

and

5 $$N(\rho )\le \sum {p}_{\alpha }N(|{\psi }_{\alpha }\rangle \langle {\psi }_{\alpha }|)\le \frac{1+2d}{3},$$

which proves the theorem.    ■

Proof of Theorem 2

Still we consider a pure state first. Let $\rho =|\psi \rangle \langle \psi |\in {H}_{1}^{d}\otimes {H}_{2}^{d}\otimes {H}_{3}^{d}$ be a pure quantum state. From the result in^32^, we have

6 $$\sqrt{1-tr{\rho }_{1}^{2}}\ge \frac{1}{\sqrt{d(d-1)}}(||{\rho }^{{T}_{1}}||-1);$$

7 $$\sqrt{1-tr{\rho }_{2}^{2}}\ge \frac{1}{\sqrt{d(d-1)}}(||{\rho }^{{T}_{2}}||-1);$$

8 $$\sqrt{1-tr{\rho }_{3}^{2}}\ge \frac{1}{\sqrt{d(d-1)}}(||{\rho }^{{T}_{3}}||-1).$$

One computes

$$\begin{array}{lll} &  & 3\sqrt{d(d-\mathrm{1)}}\sqrt{1-tr{\rho }_{1}^{2}}-3\,{\rm{\max }}\,\{M(\rho ),N(\rho )\}+1+2d\\  & = & 3\sqrt{d(d-\mathrm{1)}}\sqrt{1-tr{\rho }_{1}^{2}}-(||{\rho }^{{T}_{1}}||+||{\rho }^{{T}_{2}}||+||{\rho }^{{T}_{3}}||)+1+2d\\  & \ge  & 3\sqrt{d(d-\mathrm{1)}}\sqrt{1-tr{\rho }_{1}^{2}}-\sqrt{d(d-\mathrm{1)}}(\sqrt{1-tr{\rho }_{1}^{2}}+\sqrt{1-tr{\rho }_{2}^{2}}+\sqrt{1-tr{\rho }_{3}^{2}})-2+2d\\  & = & 2\sqrt{d(d-\mathrm{1)}}\sqrt{1-tr{\rho }_{1}^{2}}-\sqrt{d(d-\mathrm{1)}}(\sqrt{1-tr{\rho }_{2}^{2}}+\sqrt{1-tr{\rho }_{3}^{2}})-2+2d\\  & \ge  & 2\sqrt{d(d-\mathrm{1)}}\sqrt{\frac{d-1}{d}}-2+2d=\mathrm{0,}\end{array}$$

where we have used $\sqrt{1-tr{\rho }_{1}^{2}}\ge 0$ and $\sqrt{1-tr{\rho }_{k}^{2}}\le 1-\frac{1}{d}$, *k* = 2 or 3 to obtain the last inequality above.

Thus we get

9 $$\sqrt{1-tr{\rho }_{1}^{2}}\ge \frac{1}{\sqrt{d(d-1)}}(\max \,\{M(\rho ),N(\rho )\}-\frac{1+2d}{3}).$$

Similarly we obtain

10 $$\sqrt{1-tr{\rho }_{2}^{2}}\ge \frac{1}{\sqrt{d(d-1)}}(\max \,\{M(\rho ),N(\rho )\}-\frac{1+2d}{3}).$$

11 $$\sqrt{1-tr{\rho }_{3}^{2}}\ge \frac{1}{\sqrt{d(d-1)}}(\max \,\{M(\rho ),N(\rho )\}-\frac{1+2d}{3}).$$

Then according to the definition of GME concurrence, we derive

12 $${C}_{GME}(|\psi \rangle )\ge \frac{1}{\sqrt{d(d-1)}}(\max \,\{M(\rho ),N(\rho )\}-\frac{1+2d}{3}).$$

Now we consider a mixed state $\rho \in {H}_{1}^{d}\otimes {H}_{2}^{d}\otimes {H}_{3}^{d}$ with the optimal ensemble decomposition *ρ* = ∑_*α*_ *p*_*α*_|*ψ*_*α*_〉〈*ψ*_*α*_| s.t. the GME concurrence attains its minimum. One gets

$$\begin{array}{rcl}{C}_{GME}(\rho ) & = & \sum _{{p}_{\alpha },|{\psi }_{\alpha }\rangle }{p}_{\alpha }{C}_{GME}(|{\psi }_{\alpha }\rangle )\\  & \ge  & \frac{1}{\sqrt{d(d-1)}}(\max \,\{M(\rho ),N(\rho )\}-\frac{1+2d}{3})\sum _{\alpha }{p}_{\alpha }\\  & = & \frac{1}{\sqrt{d(d-1)}}(\max \,\{M(\rho ),N(\rho )\}-\frac{1+2d}{3})\end{array}$$

which ends the proof of the theorem.    ■
